# Thermal Conductivity of UO_2_ with Defects via DFT+U Calculation and Boltzmann Transport Equation

**DOI:** 10.3390/ma18153584

**Published:** 2025-07-30

**Authors:** Jiantao Qin, Min Zhao, Rongjian Pan, Aitao Tang, Lu Wu

**Affiliations:** 1 The First Sub-Institue, Nuclear Power Institute of China, Chengdu 610005, China; qjtuestc@163.com (J.Q.); zhaomts@163.com (M.Z.); haoyunjiuzhe2008@126.com (R.P.); 2 College of Material Science and Engineering, Chongqing University, Chongqing 400044, China; tat@cqu.edu.cn; 3 National Key Laboratory of Nuclear Reactor Technology, Nuclear Power Institute of China, Chengdu 610041, China; 4 State Key Laboratory of Advanced Nuclear Energy Technology, Nuclear Power Institute of China, Chengdu 610213, China

**Keywords:** phonon, thermal conductivity, irradiation defects, scattering, DFT, Boltzmann transport equation

## Abstract

Accurate evaluation of the thermal conductivity of UO_2_ with defects is very significant for optimizing fuel performance and enhancing the safety design of reactors. We employed a method that combines the Boltzmann transport equation with DFT+U to calculate the thermal conductivity of UO_2_ containing fission products and irradiation-induced point defects. Our investigation reveals that the thermal conductivity of UO_2_ is influenced by defect concentration, defect type, and temperature. Fission products and irradiation defects result in a decrease in thermal conductivity, but they have markedly different impacts on phonon scattering mechanisms. Metal cations tend to scatter low-frequency phonons (less than 5.8 THz), while the fission gas xenon scatters both low-frequency and high-frequency phonons (greater than 5.8 THz), depending on its occupancy at lattice sites. Uranium vacancies scatter low-frequency phonons, while oxygen vacancies scatter high-frequency phonons. When uranium and oxygen vacancies coexist, they scatter phonons across the entire frequency spectrum, which further results in a significant reduction in the thermal conductivity of UO_2_. Our calculated results align well with experimental data across a wide temperature range and provide fundamental insights into the heat transfer mechanisms in irradiated UO_2_. These findings are essential for establishing a thermal conductivity database for UO_2_ under various irradiation conditions and benefit the development of advanced high-performance UO_2_ fuel.

## 1. Introduction

Uranium dioxide (UO_2_) is widely used as a nuclear fuel in reactors due to its high melting point (~2800 °C), excellent chemical stability, superior radiation resistance, and good compatibility with coolants [1]. However, as a ceramic fuel, UO_2_ suffers from a relatively low thermal conductivity (only around 8~10 W/(mK) at room temperature [2]). During operating conditions, the collision between fission fragments and lattice atoms will generate numerous point defects (e.g., vacancy-interstitial pairs). Most of these defects undergo recombination or migrate to sinks, such as dislocations and stacking faults, leaving only a small fraction stable [3]. Simultaneously, the fission of ^235^U produces a variety of solid fission products (e.g., Mo, Ru, Ba) that either precipitate as metals or dissolve as metal oxides, along with gaseous fission products (e.g., Xe, Kr) dispersed within the UO_2_ matrix [4]. These irradiation-induced defects and fission products introduce lattice distortion and stress concentration into the matrix, disrupting the translational symmetry and periodicity of the lattice and acting as scattering centers for heat transport. Since UO_2_ is a Mott insulator [5], heat conduction primarily occurs through phonons. However, radiation defects, along with solid and gaseous fission products scatter phonons, thereby reducing the phonon mean free path (MFP). The scattering strength of these point defects is proportional to the fourth power of the phonon frequency [6]. Furthermore, as burnup increases, the accumulation of fission products leads to the formation of a “high burnup structure” (HBS), which enhances phonon scattering, introduces thermal resistance phases (e.g., bubbles), significantly degrades the thermal conductivity of UO_2_ [7], and can result in pellet melting and fission gas release. Therefore, accurately predicting the thermal conductivity of irradiated UO_2_ is crucial for reactor safety and further performance optimization.

Thermal transport in nuclear fuels has been extensively investigated through both experimental and theoretical calculations. Martin et al. [8] measured the thermal diffusivity (α), heat capacity ($C_{P}$), and density (ρ) of non-stoichiometric and porous UO_2_. They fitted the empirical thermal conductivity formula $\kappa =\alpha C_{P}\rho$ with measured data using a first-order phonon scattering model, represented as $\alpha^{-1}=A+BT$. In this model, parameters A and B correlate with phonon-defect and phonon-phonon scattering, respectively, while T represents temperature, thereby laying the groundwork for further experimental research. Minato et al. [9] employed the laser flash method to measure the thermal conductivity of disk-shaped UO_2_ and (U,Gd)O_2_ samples, demonstrating that irradiation-induced point defects significantly reduce the thermal conductivity of UO_2_. Philipponneau et al. [10] measured the thermal conductivity of non-stoichiometric and porous (U,Pu)O_2−x_ mixed oxide (MOX) fuels and established a new empirical formula. Ronchi et al. [11] extended Martin’s model by incorporating irradiation effects, developing a unified empirical formula that describes the thermal conductivity of both pristine and irradiated UO_2_. This model provides a precise characterization of irradiation temperature and burnup, enabling accurate predictions of the thermal conductivity for UO_2_ under operation conditions.

Experimental measurement of thermal conductivity for irradiated UO_2_, particularly at high temperatures, remains challenging due to complex procedures and safety risks [11,12,13,14,15]. Theoretically, first-principles calculations combined with Slack theory [16], the Callaway model [17,18], or the phonon Boltzmann transport equation (BTE) can predict thermal conductivity. In contrast, molecular dynamics (MD) simulations [19,20,21] offer complementary insights, though they exhibit strong sensitivity to interatomic potentials, where inadequate potential models can introduce inaccuracies in thermal conductivity estimation. Currently, the thermal conductivity of irradiated UO_2_ is primarily established through experiments, which are used to create quantitative relationships with defect concentrations. However, the fundamental effects of point defects, fission products, dislocations, and grain boundaries on thermal conductivity have not been fully addressed. Although significant efforts have been made in multiscale modeling and experimental characterization, challenges persist in developing predictive models and advanced experimental tools.

In this work, we employ first-principles calculations combined with the phonon BTE to investigate the thermal conductivity of uranium dioxide (UO_2_) containing fission products and irradiation defects. This paper is organized as follows: (1) Introduction to the theory of thermal conductivity and the methods used in the calculations; (2) Results and Discussion of the electronic structure and phonon spectrum of UO_2_; (3) Thermal Conductivity of UO_2_ and a discussion of the effects of fission products and irradiation defects; (4) Conclusions and summary.

## 2. Theory and Methods

### 2.1. Theory of Thermal Conductivity

Thermal transport in insulators is primarily governed by quantized energy of lattice vibrations, known as phonons [22], and the contribution of a frequency-controlled phonon mode, denoted as ω, is determined by three fundamental parameters [6]: (1) the heat capacity $C_{v}$ of the phonon; (2) the group velocity $\upsilon_{g}=d\omega /dq$; and (3) the lifetime of the phonon τ. Accurately determining lattice thermal conductivity requires solving the phonon Boltzmann transport equation under non-equilibrium conditions. According to lattice vibartions, we can calculate the population of the phonon mode frequency $\omega_{q\nu }$, where it follows Bose–Einstein statistics under equilibrium: ${\bar{n}}_{q\nu }=1/\left(e^{\hbar \omega_{q\nu }/k_{B}T}-1\right)$. In this equation, *q*, ν, *ℏ*, and $k_{B}$ denote the wave vector, the branch index, the reduced Planck constant, and the Boltzmann constant, respectively. When the lattice is subjected to a temperature gradient ∇T, the phonon distribution deviates from equilibrium ($n_{q\nu }\neq {\bar{n}}_{q\nu }$), resulting in a phonon flux from higher to lower temperature regions. The steady-state distribution is described by the BTE [22]:

(1) $$-\frac{\partial n_{q\nu }}{\partial T}v_{q\nu }\nabla T=-\frac{\partial n_{q\nu }}{\partial t}{|}_{scatt}$$

where the left term corresponds to the drift component induced by ∇T, and the right term accounts for various scattering processes. Here, $v_{q\nu }$ denotes the group velocity of the phonon mode qν. Within the relaxation time approximation (RTA), the detailed derivation is presented in Appendix A, and the lattice thermal conductivity is expressed as [23]:

(2) $$\kappa =\frac{\hbar^{2}}{N_{0}Vk_{B}T^{2}}\sum_{q\nu }v_{q\nu }^{2}\omega_{q\nu }^{2}{\bar{n}}_{q\nu }\left({\bar{n}}_{q\nu }+1\right)\tau_{q\nu }$$

where *V* is the volume of the primitive cell, and $N_{0}$ corresponds to the number of sampling points in the q-point grid across the Brillouin zone, the total relaxation time $\tau_{q\nu }$ incorporates all scattering mechanisms according to Matthiessen’s rule.

(3) $${\left(\tau_{q\nu }\right)}^{-1}={\left(\tau_{q\nu }^{ph}\right)}^{-1}+{\left(\tau_{q\nu }^{defects}\right)}^{-1}+{\left(\tau_{q\nu }^{others}\right)}^{-1}$$

Cumulative thermal conductivity $\kappa^{c}\left(\omega \right)$ and thermal conductivity κ(ω) with respect to frequency are defined by

(4) $$\kappa^{c}\left(\omega \right)=\int_{0}^{\omega }\frac{1}{N_{0}}\sum_{q\nu }\kappa_{q\nu }\delta \left(\omega_{q\nu }-\omega^{\prime }\right)d\omega^{\prime }$$

(5) $$\kappa \left(\omega \right)=\frac{1}{N_{0}}\sum_{q\nu }\kappa_{q\nu }\delta \left(\omega_{q\nu }-\omega \right)$$

The three-phonon scattering rate is calculated using Fermi’s golden rule [24]:

(6) $$\begin{array}{cc} {\left(\tau_{q\nu }^{ph}\right)}^{-1} & =\frac{\pi }{\hbar^{2}}\sum_{q^{\prime }\nu^{\prime },q^{\prime \prime }\nu^{\prime \prime }}{\left|\Phi_{q\nu ,q^{\prime }\nu^{\prime },q^{\prime \prime }\nu^{\prime \prime }}\right|}^{2}\{\left(n_{q^{\prime }\nu^{\prime }}+n_{q^{\prime \prime }\nu^{\prime \prime }}+1\right)\delta \left(\omega_{q\nu }-\omega_{q^{\prime }\nu^{\prime }}-\omega_{q^{\prime \prime }\nu^{\prime \prime }}\right)\\ \; & +\left(n_{q^{\prime }\nu^{\prime }}-n_{q^{\prime \prime }\nu^{\prime \prime }}\right)\left[\delta \left(\omega_{q\nu }+\omega_{q^{\prime }\nu^{\prime }}-\omega_{q^{\prime \prime }\nu^{\prime \prime }}\right)-\delta \left(\omega_{q\nu }-\omega_{q^{\prime }\nu^{\prime }}+\omega_{q^{\prime \prime }\nu^{\prime \prime }}\right)\right]\} \end{array}$$

where $\Phi_{q\nu ,q^{\prime }\nu^{\prime },q^{\prime \prime }\nu^{\prime \prime }}$ represents the third-order anharmonic scattering strength matrix elements. For defect scattering, we employ the generalized Tamura model [25]:

(7) $${\left(\tau_{q\nu }^{defects}\right)}^{-1}=\frac{V_{0}\eta \omega_{q\nu }^{4}}{4\pi {\bar{v}}^{3}}$$

where $V_{0}$ is the unit cell volume, $\bar{v}$ is the average velocity of the phonons, and η is the scattering parameter [15,26,27]:

(8) $$\eta =\sum_{i}f_{i}\left[{\left(\frac{\Delta M}{\bar{M}}\right)}^{2}+2{\left(\frac{\Delta K}{\bar{K}}-2Q\gamma \frac{\Delta R}{\bar{R}}\right)}^{2}\right]$$

Here, $f_{i}$ denotes the concentration of defect type *i*, and ΔM and $\bar{M}$ represent the mass difference and average mass, respectively. Similarly, ΔK and $\bar{K}$ indicate the differences in force constants and the average force constants, while ΔR and $\bar{R}$ correspond to the differences in radius and the average radius, respectively. The average Grüneisen parameter is denoted as γ. The parameter *Q*, which is 4.2 for vacancies and 3.2 for substitutional defects in fluorite structures [28], accounts for nearest-neighbor bond distortion. The variation in force constants is typically proportional to volume, merging the combination of force constant and radius variation terms as follows:

(9) $$\eta =\sum_{i}f_{i}\left[{\left(\frac{\Delta M}{\bar{M}}\right)}^{2}+\varepsilon {\left(\frac{\Delta R}{\bar{R}}\right)}^{2}\right]$$

The dimensionless parameter ε (typically ranging from 0 to 500) is determined through empirical fitting. There are three distinct defect scattering regimes [29]: (a) Mass-only defects (e.g., isotopic impurities): Only the mass variance term in Equation (9) contributes significantly; (b) Substitutional impurities (e.g., Mo in UO_2_): Both mass and volume terms are relevant, with possible bond modifications; (c) Vacancies/interstitials: All terms contribute substantially, including significant modifications to the force constants. For noble gas atoms (Xe, Kr) that do not form chemical bonds with the matrix, the scattering is dominated by mass and volume effects, with minimal contributions from force constants. This comprehensive approach facilitates accurate modeling of phonon scattering by various defect types in nuclear fuel materials.

### 2.2. Methods

**Figure 1:**
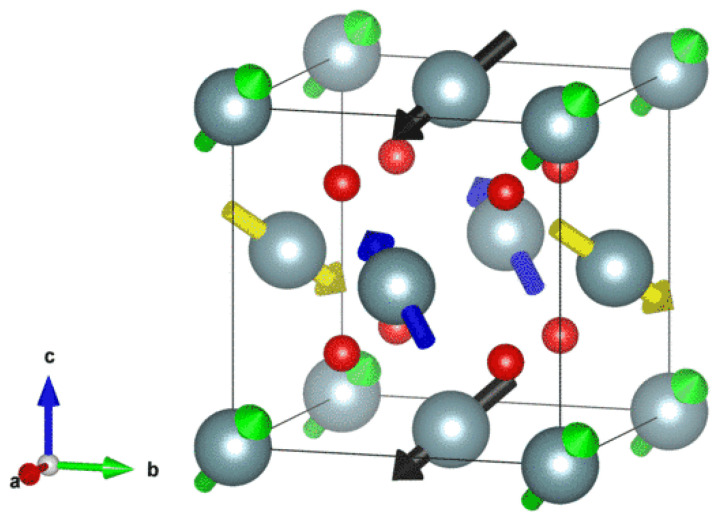
The 3k antiferromagnetic (AFM) ordering in the conventional unit cell of UO_2_, where the fractional coordinates of the U atoms are (0, 0, 0), (0, 0.5, 0.5), (0.5, 0, 0.5), and (0.5, 0.5, 0), with the corresponding magnetic moments being (1, 1, 1), (−1, −1, 1), (−1, 1, −1), and (1, −1, −1) in units of $\mu_{B}$.

First-principles calculations were performed using the Vienna Ab initio Simulation Package (VASP) within the framework of density functional theory (DFT) [30]. The projector augmented wave (PAW) method [31] was employed to describe the electron-ion interactions, and the exchange-correlation functional was treated with the generalized gradient approximation (GGA) in the Perdew-Burke-Ernzerhof (PBE) formulation [32]. To accurately account for the strong electron correlations in UO_2_, the Hubbard U correction (DFT+U) [33] was applied, incorporating spin-orbit coupling (SOC) to improve the accuracy of the electronic structure, with the spin quantization axis aligned along the z-axis. The initial structure of UO_2_ was modeled using a conventional unit cell (containing 4 U and 8 O atoms), as illustrated in Figure 1. A 2 × 2 × 2 supercell (96 atoms) was constructed with the 3k antiferromagnetic (AFM) ordering [34,35] along the a, b, and c axes. The plane-wave cutoff energy was set to 450 eV, and a 3 × 3 × 3 Monkhorst-Pack k-point mesh was used for Brillouin zone sampling, and the total energy convergence criterion was set to 10^−6^ eV. Structural optimization was performed under zero external pressure to obtain the equilibrium lattice parameters. The f-orbital occupation matrix control (OMC) method [36] was adopted to avoid metastable states and accelerate iterative convergence.

Subsequently, in order to accurately describe thermal transport in lattice, second- and third-order interatomic force constants (IFCs) were calculated by DFT+U and the supercell structures of UO_2_ were generated by the finite displacement method as implemented in the Phonopy [37] and Phono3py [38] codes with atomic displacements of 0.03 Å in supercell. The Born effective charges, dielectric constant tensor of UO_2_ were calculated by density functional perturbation theory (DFPT) [39] and the long-range Coulomb interactions in ionic crystal of UO_2_ (U^4+^ and O^2−^) were corrected by introducing non-analytical term corrections (NAC) [40]. Finally, the lattice thermal conductivity ($\kappa_{l}$) of UO_2_ was computed by solving the Boltzmann transport equation within the relaxation time approximation. A 19 × 19 × 19 q-point mesh was used for Brillouin zone integration, and the tetrahedron method was applied to improve numerical accuracy. Symmetry operations were utilized to reduce computational costs and maintain high precision.

## 3. Results and Discussions

### 3.1. Electronic Structure

**Figure 2:**
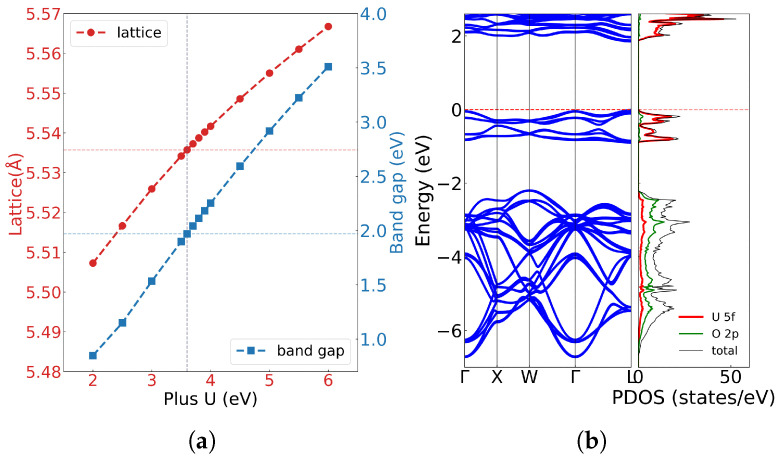
(**a**) The lattice constant of UO_2_ with the U parameter; (**b**) the band structure and electronic density of states of UO_2_ at U = 3.60 eV and J = 0.0 eV.

First-principles calculations provide fundamental insights into the electronic structure of uranium dioxide. Figure 2a illustrates the lattice constants and bandgap ($E_{g}$) as a function of the Hubbard U with spin-orbit coupling. Both lattice constant and bandgap increase nearly linearly with U values, consistent with previous calculations by Dorado et al. [36]. Experimentally, UO_2_ exhibits a lattice constant of a = c = 5.47 Å, and the bandgap is approximately 2.0 eV. By comparing our result with experiment, a parameter of U = 3.6 eV and J = 0.0 eV was selected in our calculation, which can yield a bandgap of 1.990 eV, in excellent agreement with the experimental value of 2.0 eV. As shown in Figure 2b, the calculated band structure and density of state reveal that the valence band maximum and conduction band minimum are primarily derived from U 5f and O 2p orbitals.

Within U = 3.6 eV and J = 0.0 eV, our calculated lattice constant was a = c = 5.536 Å, showing consistency with previous studies. While Dorado et al. [36] obtained the lattice parameter of a = 5.56 Å and c = 5.50 Å (symmetry breaks and a≠c) with U = 4.50 eV and J = 0.51 eV according to the experimental elastic constant. A similar c-axis contraction was also observed by Iwasawa et al. [41] and may be attributed to spin alignment in the antiferromagnetic (AFM) state, where U atoms with opposite magnetic moments move closer along the *z* axis. Meanwhile, using the PBE functional with U = 3.35 eV and J = 0.0 eV, Pegg et al. [34] found that the lattice constant a = c = 5.474 Å and the magnetic moment was insensitive to U or J. These results confirm that the PBE functional with Hubbard U correction can provide an accurate electronic structure but tends to overestimate the lattice parameter. All these computational results conclusively demonstrate that UO_2_ exhibits Mott insulating behavior where valence electrons are strongly localized around lattice atoms. This electronic configuration makes lattice vibrations (phonon) the dominant factor in heat transfer, while electronic thermal conductivity ($\kappa_{e}$) only manifests a negligible influence.

### 3.2. Phonon Spectrum

**Figure 3:**
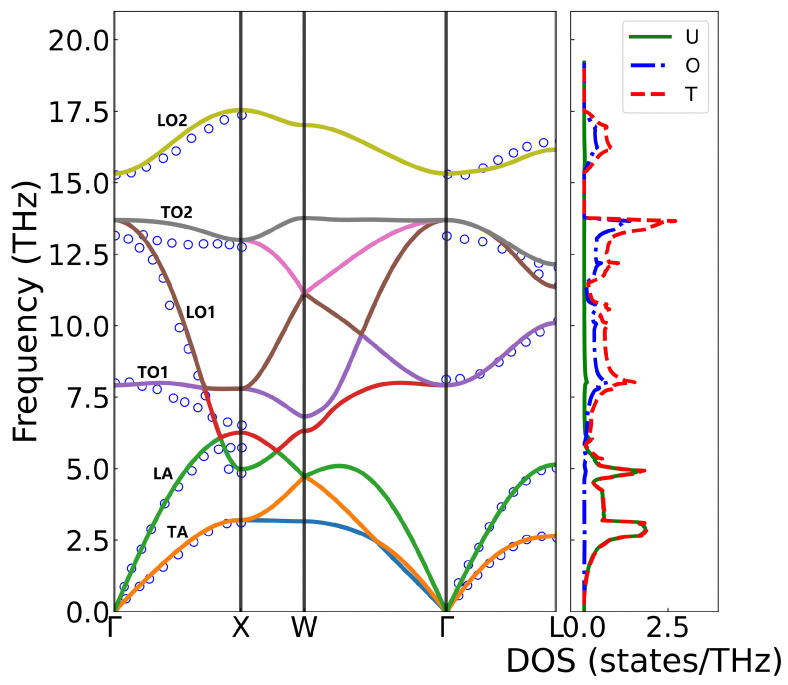
The phonon spectrum and density of states of UO_2_. The hollow circles represent the measured values from inelastic neutron scattering, and the solid lines represent the simulated DFT+U calculation results. The fractional coordinates of the high-symmetry points in the Brillouin zone are Γ(0 0 0), X (0.5 0 0.5), W (0.5 0.25 0.75), and L (0.5 0.5 0.5).

The phonon spectrum and density of states of UO_2_ were investigated using first-principles calculations and compared with experimental data from inelastic neutron scattering measurements at 296K [42,43], as shown in Figure 3. UO_2_ crystallizes in the fluorite structure (Fm3-m), with a primitive cell containing one uranium atom and two inequivalent oxygen atoms, giving rise to nine phonon branches—three acoustic (TA/LA) and six optical (TO/LO). The calculated phonon dispersion along the high-symmetry Γ-X-W-Γ-L path agrees well with experimental results, except for the TO2 branch near the Γ point, where the computed frequencies are slightly higher, likely due to the absence of temperature effects in the 0 K simulations. The acoustic modes, dominated by uranium vibrations, appear at low frequencies, while the optical branches, primarily associated with oxygen motion, exhibit higher frequencies. Obviously, longitudinal optical (LO2) and transverse optical (TO2) phonon branches split at the Γ point due to the long-range Coulomb interaction, which was accounted for by incorporating a non-analytical term in the dynamical matrix. The diagonal element of the static dielectric tensor yielded 5.4512, while the Born effective charge was calculated as $Z_{U}^{*}=+5.0378$ and $Z_{O}^{*}=-2.5189$, indicating significant charge transfer and partial covalent character.

The maximum phonon frequency depicted in the figure occurs at the X-point, with a value of 17.54 THz. Near the Γ-point, with the exception of the TO2 branch, the calculated phonon frequencies demonstrate excellent agreement with experimental measurements. Along the Γ-X high-symmetry path, slight discrepancies exist between the calculations and experimental measurement, with the calculated results exhibiting higher values than the experimental data. This difference may arise because the experimental measurements were conducted at 296 K, while the calculations were performed at 0 K; increasing temperature typically leads to phonon frequency softening. For the LA and TA branches, the calculated values align well with experimental measurements, although minor differences are observed at the boundary X-point. No direct comparison was made for frequencies between the X and W points due to the different paths taken in the experiment and calculation. Along the Γ-L path, the calculations and experimental results show good agreement. However, a relatively larger deviation is noted for the highest-frequency optical branch (TO2), which may be attributed to greater experimental uncertainties in measuring high-frequency optical modes.

The maximum phonon frequency in the figure occurs at the X-point with a value of 17.54 THz. Near the Γ-point, with the exception of the TO2 branch, the calculated phonon frequencies show excellent agreement with experimental measurements. Along the Γ-X high-symmetry path, slight discrepancies exists between calculation and experimental measurement and the calculated result exhibits higher value than experimental data. This difference may arise because the experimental measurement was performed at 296 K while calculations at 0 K, as increasing temperature typically leads to phonon frequency softening. For the LA and TA branches, the calculated values match well with experimental measurements, though minor differences appear at the boundary X-point. No direct comparison was made for frequencies between the X and W points due to different paths taken in experiment and calculation. Along the Γ-L path, the calculations and experimental results show good agreement. However, a relatively larger deviation is observed for the highest-frequency optical branch (TO2), which may be attributed to greater experimental uncertainties in high-frequency optical modes measurement.

**Figure 4:**
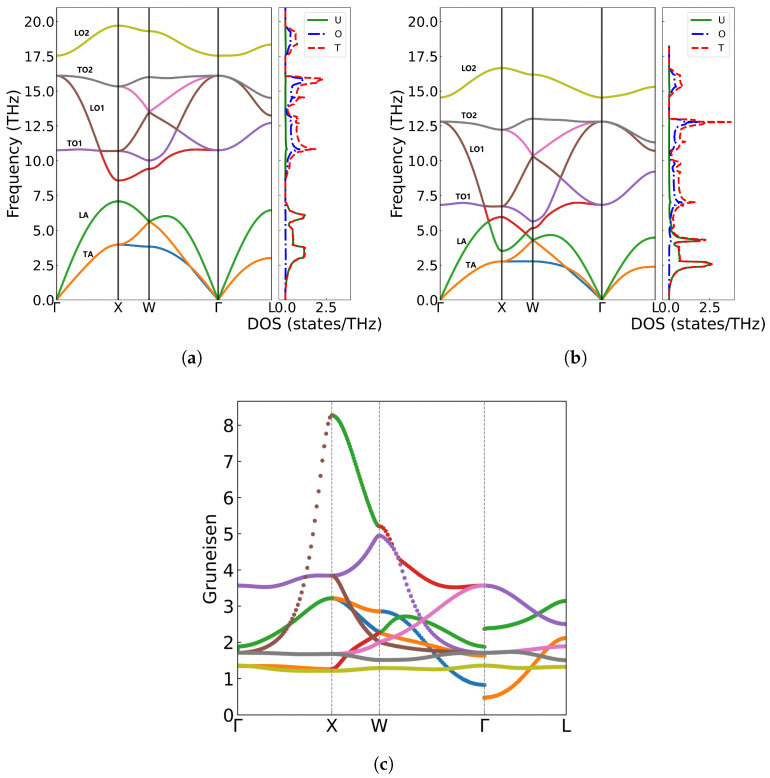
Phonon spectra, density of states, and Grüneisen parameters of UO_2_ at different volumes: (**a**) a = 5.50 Å, (**b**) a = 5.540 Å, (**c**) Grüneisen parameters along high-symmetry points in the Brillouin zone.

From Figure 4a,b, we can see that phonon frequencies exhibit a strong dependence on lattice parameter variations. Using the equilibrium lattice constant a = 5.536 Å as reference, under compression (a = 5.50 Å) phonon hardening occurs, with the maximum frequency increasing to 19.698 THz at the X point and a widening gap between the LA and TO1 branches [44,45,46]. Conversely, lattice expansion (a = 5.540 Å) leads to phonon softening, reducing the highest frequency to 16.648 THz and causing the LA and TO1 branches to overlap. Such phonon frequency shifts with volume variation in UO_2_ may originate from the anharmonic nature of interatomic potentials. Our calculations reveal distinct phonon hardening under compression (a = 5.50 Å) and softening under expansion (a = 5.540 Å), demonstrating the significant role of lattice anharmonicity in vibrational properties. To quantitatively characterize these effects, we computed Grüneisen parameters (γ) along high-symmetry directions with the formula $\gamma =-\partial \left(\ln\omega \right)/\partial \left(\ln V\right)$. As shown in Figure 4c, our calculated Grüneisen parameters exhibit notable branch dependence, with an average value of 1.86, consistent with both theoretical [47] (1.88 from PBE+U calculations) and experimental (1.6–2.2) values.

### 3.3. Thermal Conducitivity

#### 3.3.1. Pristine UO_2_

**Figure 5:**
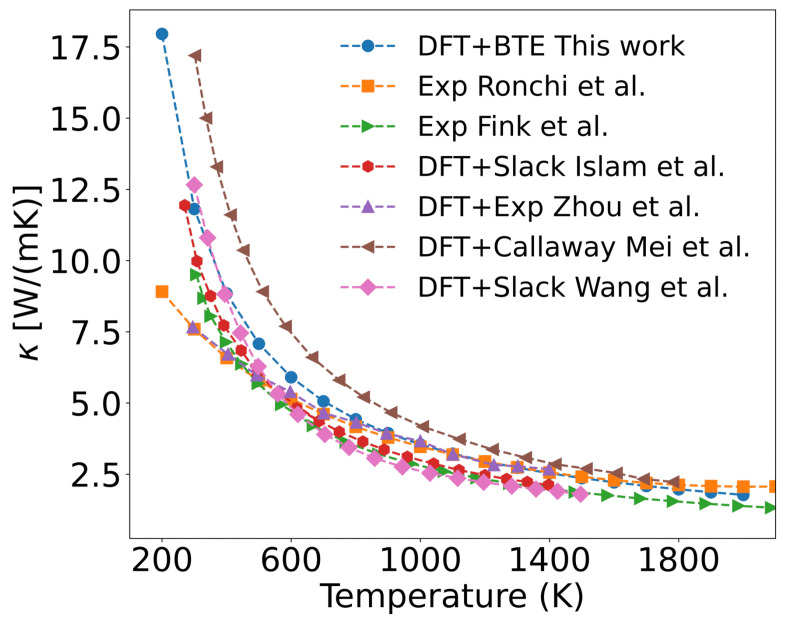
Calculated phonon thermal conductivity of UO_2_ using the DFT+U+SOC method (filled circle), compared with experimental measurements and theoretical predictions from literature. The comparison includes: square for Ronchi et al. [48], rightward triangle for Fink et al. [49], filled hexagon for Islam et al. [50], upward triangle for Zhou et al. [51], leftward triangle for Mei et al. [52], and diamonds for Wang et al. [16].

The thermal conductivity of unirradiated UO_2_, calculated through first-principles simulation combined with the Boltzmann transport equation, is shown in Figure 5. Since the UO_2_ single crystal contains no defects, its thermal conductivity is primarily governed by three-phonon scattering. As observed in the figure, the thermal conductivity gradually decreases with increasing temperature. At low temperatures (e.g., 300 K), lattice vibrations remain close to equilibrium positions, satisfying the harmonic approximation well. Under these conditions, different phonon modes are nearly independent, and phonon-phonon scattering is weak, resulting in high thermal conductivity (11.813 W/(m·K) at 300 K). As temperature rises, atomic vibrations deviate further from equilibrium, and the interatomic potential becomes asymmetric, breaking the independence of phonon modes. This leads to enhanced phonon scattering, such as the merging of two phonons into one or the decay of one phonon into two. Three-phonon scattering includes Normal (N) processes and Umklapp (U) processes. While N processes only redistribute phonon momentum without affecting thermal conductivity, U processes cause significant momentum reversal, increasing phonon scattering and reducing the mean free path. Consequently, thermal conductivity drops sharply at high temperatures (e.g., 1.970 W/(m·K) at 1800 K).

Figure 5 shows the calculated thermal conductivity of a perfect UO_2_ crystal only considering three-phonon scattering. The results exhibit a similar variation trend with existing results. In experiment, Ronchi [48], Fink [49], and others experimentally determined the thermal diffusivity, heat capacity, and density of UO_2_, from which they derived empirical formulas for thermal conductivity. The measured thermal conductivity in UO_2_ is lower than our calculated values in the 200–600 K range, as shown in Figure 5. This discrepancy may be ascribed to that UO_2_ samples used in experiments contain various impurities and defects (such as vacancies, grain boundaries, and surfaces). At lower temperatures, these defects scatter phonons, leading to a reduction of thermal conductivity in UO_2_ samples compared to the perfect single crystal. A detailed analysis of this effect will be presented later.

To validate our results, Figure 5 also presents several calculated thermal conductivity values of UO_2_. Among these, using Slack’s empirical model, Islam [50] and Wang [16] calculated the Debye temperature, Grüneisen parameter, and thermal conductivity. Their results, as shown in Figure 5, agree well with our calculations. However, this model cannot account for the thermal conductivity of defective UO_2_. Zhou [51] incorporated thermal expansion effects and employed first-principles calculations combined with the phonon Green’s function, using experimentally measured phonon linewidths for thermal conductivity calculations. Their results were lower and closer to experimental values. In contrast, Mei [52] applied the Callaway model with relaxation time approximation while adding a phonon momentum conservation term, leading to an overestimation of thermal conductivity compared to experimental data.

**Figure 6:**
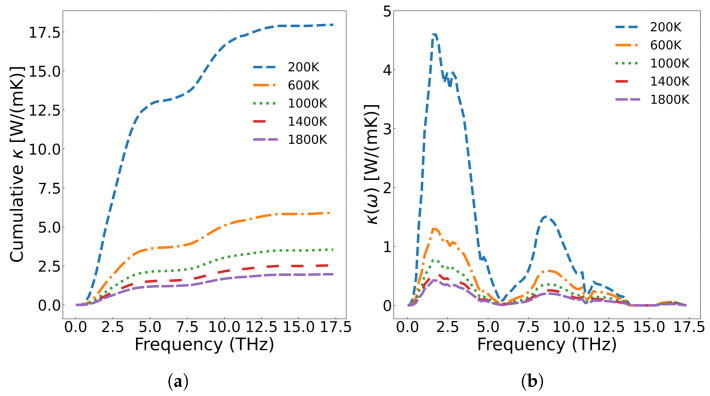
(**a**) Cumulative thermal conductivity of UO_2_ at different temperatures; (**b**) the thermal conductivity of UO_2_ with respect to phonon frequency.

**Figure 7:**
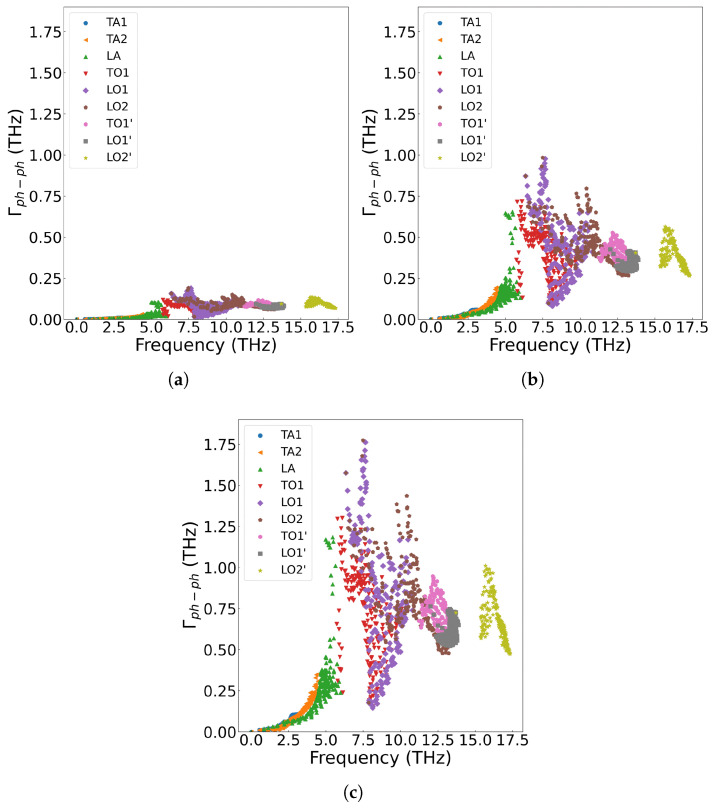
Three-phonon scattering strength in single-crystal UO_2_ at different temperatures: (**a**) 200 K (**b**) 1000 K, (**c**) 1800 K.

Figure 6a displays the frequency-dependent curve of cumulative thermal conductivity. In the figure, the contribution of phonons with different vibration frequencies is fully analyzed. As shown in Figure 6a, cumulative thermal conductivity grows rapidly as phonon frequency is below 5.8 THz, followed by slower progression in the 5.8–14.0 THz range, and for frequencies above 14.0 THz, shows no further change. Higher temperatures result in a smaller cumulative thermal conductivity value, and the variation of the cumulative thermal conductivity becomes more gradual at elevated temperatures. Figure 6b presents the thermal conductivity varying with respect to frequency. From the figure, we can see that acoustic phonons exhibit a significantly higher contribution to thermal conductivity compared with optical phonons. According to Equation (7), the thermal conductivity is multiplied by the phonon density of states. The analysis reveals that thermal conductivity is primarily governed by low-frequency (less than 5.8 THz) acoustic phonons, followed by mid-frequency optical phonons, while the high-frequency LO2’ optical branch shows negligible contribution. Additionally, the thermal conductivity decreases with increase of temperature across all frequency ranges. Figure 7 displays the three-phonon scattering strength for different phonon frequency branches at various temperatures. The results indicate that phonon scattering becomes more pronounced across all frequency branches as temperature increases, leading to a reduction in the mean free path and a shortening of phonon lifetimes.

#### 3.3.2. Solid Fission Products

**Figure 8:**
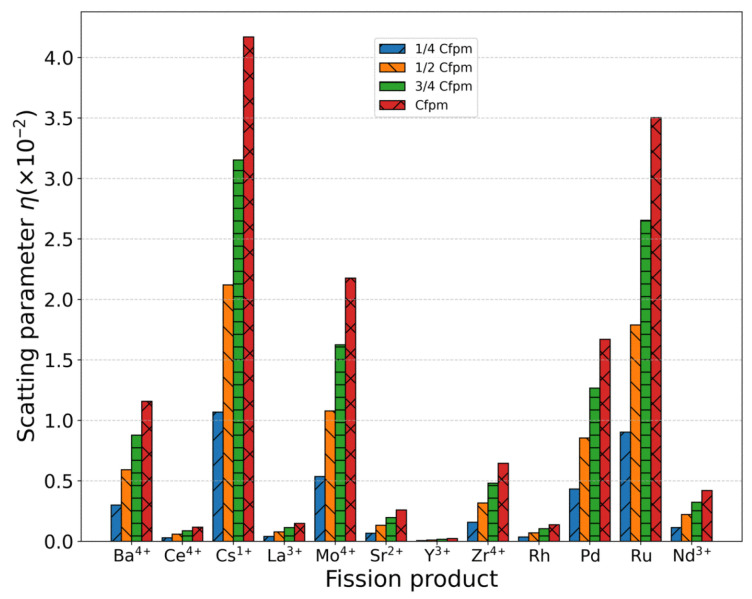
Scattering parameter η of metal ions/atoms with different mass and radii under each concentration.

The metallic elements produced during nuclear fission predominantly exist as cationic species [4,53]. These metallic atoms incorporate substitutionally into the UO_2_ lattice, replacing uranium atoms and acting as scattering centers for phonon transport. The phonon scattering strength induced by these defects can be quantified using Equation (7). Since the concentration of metal ions remains constant with temperature variation, their scattering strength depends exclusively on their concentration and intrinsic properties rather than thermal conditions. Table 1 presents the concentrations of various metallic fission products measured in SIMFUEL samples (76 GWd/tU burnup) from Chalk River Laboratories, Canada [54]. The scattering parameter η of each metallic ion was calculated based on its valence state and ionic radius within the UO_2_ matrix, with $U^{4+}$ and $O^{2-}$ reference radii of 1.001 Å and 1.368 Å, respectively, as shown in Table 1. The calculated scattering strength of fission product elements are presented in Figure 8, revealing the following descending order of scattering strength: ${\mathrm{Cs}}^{1+}$ > Ru > ${\mathrm{Mo}}^{4+}$ > Pd > ${\mathrm{Ba}}^{4+}$ > ${\mathrm{Zr}}^{4+}$ > ${\mathrm{Nd}}^{3+}$ > ${\mathrm{Sr}}^{2+}$ > ${\mathrm{La}}^{3+}$ > Rh > ${\mathrm{Ce}}^{4+}$ > $Y^{3+}$. Analysis of these point defects in Table 1 demonstrates that the scattering strength is governed by two key parameters: (1) the mass difference between impurity and host atoms ($|M_{i}-\bar{M}|$); (2) the ionic radius mismatch ($|R_{i}-\bar{R}|$). Notably, the radius difference ($|R_{i}-\bar{R}|$) dominates the scattering behavior, as it simultaneously accounts for: (1) modifications in local force constants between the dopant and surrounding matrix atoms; (2) lattice strain induced by volumetric changes at defect sites.

In Equation (9), the constant ε is typically determined through experimental measurements. Masayuki et al. [55] investigated the thermal conductivity of ceramic solid solutions in ThO_2_-UO_2_ fuel systems. By fitting thermal conductivity data across different temperatures and uranium solid solubility levels, they determined ε = 100 for the ThO_2_ system, which was subsequently used to accurately predict the thermal conductivity of ThO_2_-CeO_2_ at various temperatures. Duriez et al. [27] adopted a linear regression approach in their study of thermal conductivity in non-stoichiometric (U,Pu)O_2−x_ mixed oxide fuels with low plutonium content, obtaining ε = 25.85 for UO_2_. However, there remains no consensus on the optimal value of ε, and this study adopts ε = 25.85.

**Figure 9:**
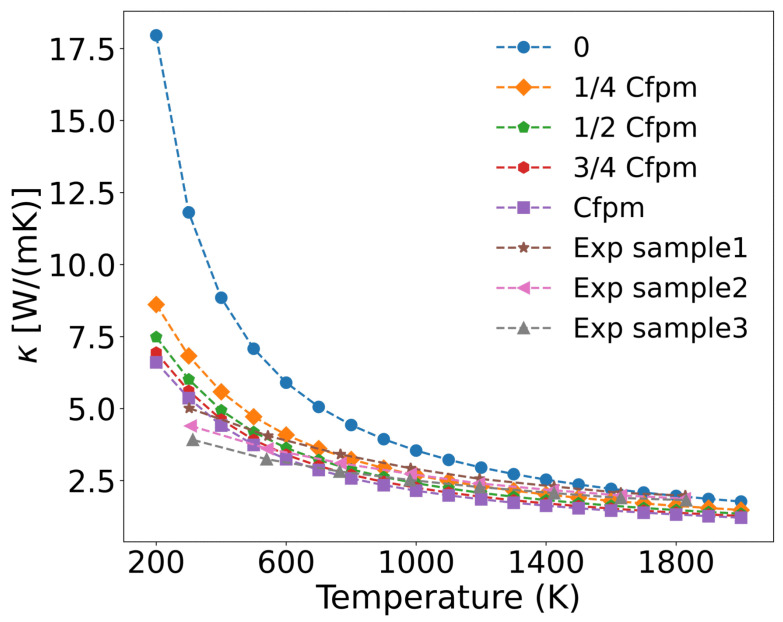
Concentration-dependent thermal conductivity of UO_2_ with fission product metal ions.

Based on measured concentrations of metallic fission products (denoted as Cfpm) in UO_2_ fuel at 76 GWd/tU burnup, and assuming a linear relationship between metal atom concentration and burnup, we established four concentrations levels: 1/4 Cfpm, 1/2 Cfpm, 3/4 Cfpm, and Cfpm, corresponding to burnups of 19 GWd/tU, 38 GWd/tU, 57 GWd/tU, and 76 GWd/tU, respectively. The fission product concentrations and burnup in UO_2_ fuel are typically determined through experimental measurements and numerical simulations [56]. The temperature-dependent thermal conductivity of UO_2_ was then calculated for these varying metal ion concentrations. As shown in Figure 9, the thermal conductivity progressively decreases with increasing metal ion concentration, and the calculated values align well with experimental data [57], which simulated burnup by doping fission product elements into UO_2_. Sample1, sample2, and sample3 correspond to simulated burnup levels of 30 GWd/tU, 60 GWd/tU, and 90 GWd/tU, respectively, with the concentrations of doping fission product elements are listed in Table 2.

The incorporation of metallic defects reduces the thermal conductivity of UO_2_, particularly in the 200–1000 K range, where reductions exceed 50%. However, the incremental effect diminishes at higher concentrations (1/4 Cfpm to Cfpm), suggesting that the impact of fission product metal ions on thermal conductivity saturates beyond a threshold concentration. All three experimental samples exhibited lower thermal conductivity than the calculated values. As shown in Table 1 and Table 2, the higher concentrations of solid fission products correlate with a greater degradation of thermal conductivity.

**Figure 10:**
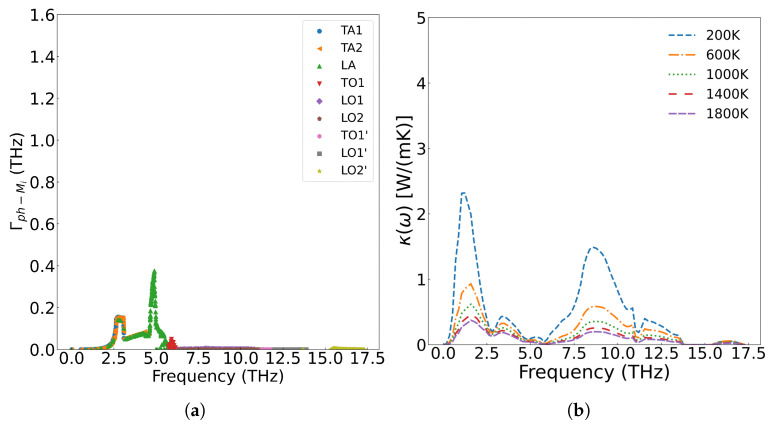

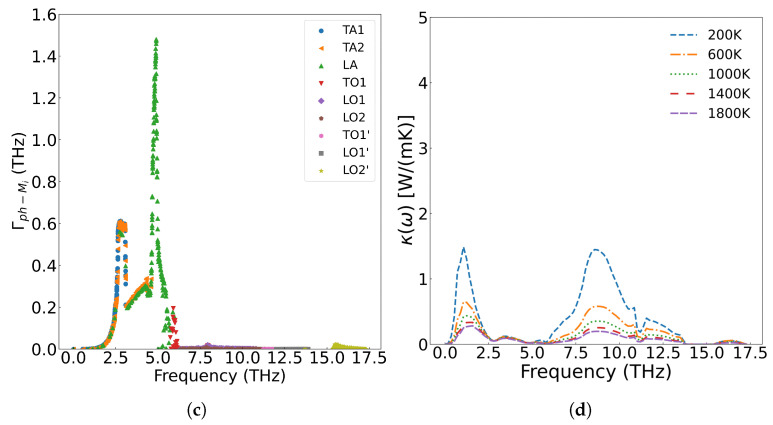
The scattering strength of metal ions on phonons at different concentrations: (**a**) 1/4 Cfpm, (**c**) Cfpm; the effect of scattering on thermal conductivity at different concentrations: (**b**) 1/4 Cfpm, (**d**) Cfpm.

Figure 10 presents the frequency-dependent phonon scattering strength. Lower defect concentrations yield weaker scattering, with metallic fission products primarily scattering the low-frequency (less than 5.8 THz) acoustic branches generated by uranium atom vibration. The scattering strength increases with concentration, while optical branches originating from oxygen atom vibrations remain virtually invariant. Consequently, the reduction in thermal conductivity predominantly stems from scattering of low-frequency phonons.

#### 3.3.3. Fission Gas Xe

**Figure 11:**
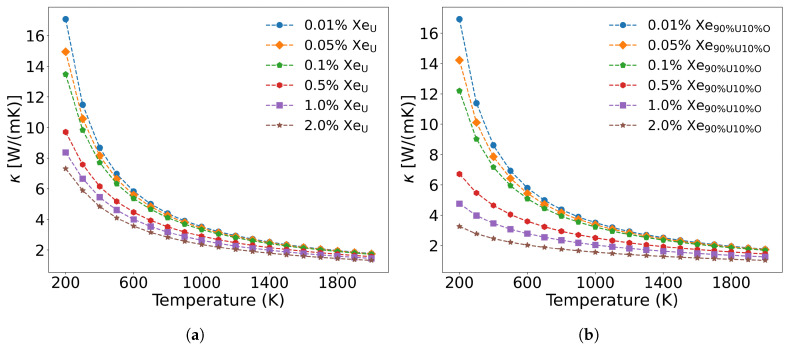
The effect of Xe concentration on thermal conductivity: (**a**) Xe substitutes only U, and (**b**) Xe simultaneously substitutes 90% U and 10% O.

The fission gas atom Xe differs from solid fission metal atoms in that, as an inert gas atom, it scarcely forms chemical bonds with the UO_2_ matrix. Its primary phonon scattering mechanisms arise from mass contrast, atomic size mismatch, and modifications to interatomic force constants near Xe defects. Xe atoms predominantly occupy irradiation-induced U and O vacancies, forming substitutional defects that are dispersed throughout the lattice. Figure 11 illustrates the influence of varying Xe concentrations on thermal conductivity when substituting for U sites alone versus simultaneous substitution at both U and O sites. As shown in Figure 11a, when Xe occupies only U vacancies, low concentrations (<0.1 at%) exhibit minimal impact on thermal conductivity, whereas higher concentrations (>0.5 at%) induce significant degradation between 200 and 1000 K. This effect diminishes at elevated temperatures (>1000 K), where three-phonon scattering becomes dominant. Below 1000 K, Xe exerts strong phonon scattering that weakens progressively with increasing temperature. These results agree with molecular dynamics simulations by CHEN et al. [58], though potentials by Basak and Busker overestimate thermal conductivity (16.10 and 18.99 W/(m·K) at 300 K compared to our calculated value of 11.813 W/(m·K). By systematically evaluating the thermal conductivity of UO_2_ at 0.01%, 0.05%, 0.1%, 0.5%, 1.0%, and 2.0% Xe concentrations, we provide fundamental data support for establishing fuel performance prediction under “high-burnup” structures.

**Figure 12:**
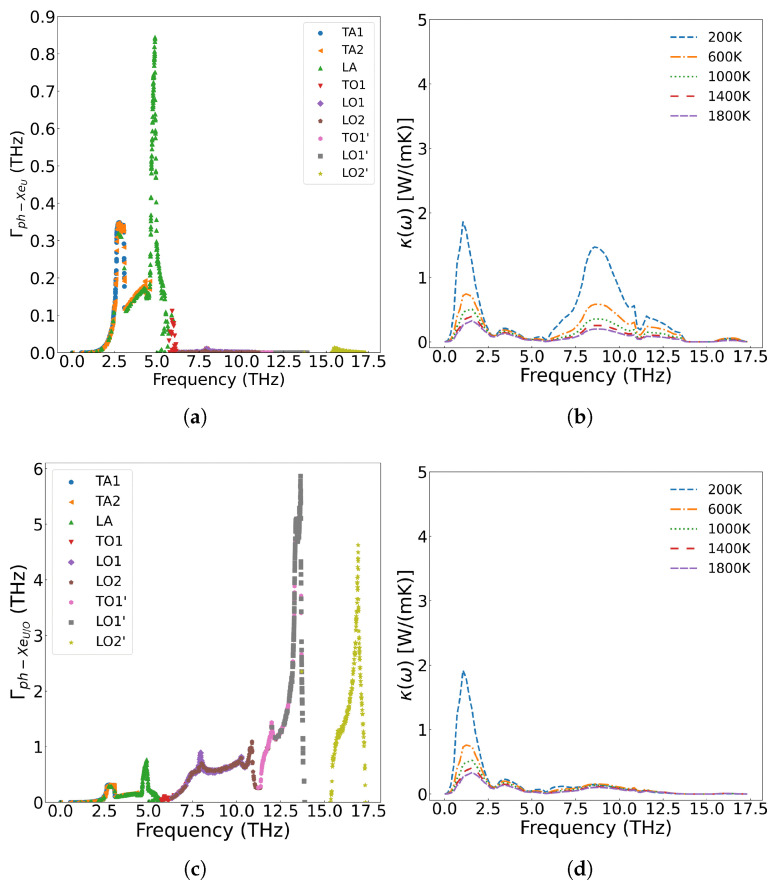
2.0 at% Xe in UO_2_: (**a**) Frequency-dependent phonon scattering strength when Xe substitutes U sites only; (**b**) Effect on thermal conductivity from U-site substitution; (**c**) Scattering strength when Xe substitutes both U and O sites; (**d**) Effect on thermal conductivity from U+O site substitution.

As shown in Figure 11a, when Xe atoms occupy only U vacancies, they primarily scatter the low-frequency (less than 5.8 THz) acoustic phonon modes generated by U atom vibrations. However, when Xe atoms simultaneously occupy both U and O vacancies at the same concentration, their impact on thermal conductivity becomes more pronounced than when occupying U vacancies alone. This is demonstrated in Figure 11b, where a distribution of 90 at% Xe in U vacancies and 10 at% Xe in O vacancies results in greater thermal conductivity reduction at identical temperatures compared to the case where Xe occupies only U sites. The underlying mechanism, illustrated in Figure 12, reveals that Xe atoms occupying O vacancies exhibit stronger scattering of high-frequency (greater than 5.8 THz) phonons (generated by O atom vibrations) than the scattering of low-frequency phonons (generated by U atom vibrations). Fission gas Xe atoms significantly degrade the thermal conductivity of UO_2_, demonstrating strong scattering effects on both low- and high-frequency phonons, with the specific influence depending on their occupation sites.

#### 3.3.4. Irradiated Point Defects

**Figure 13:**
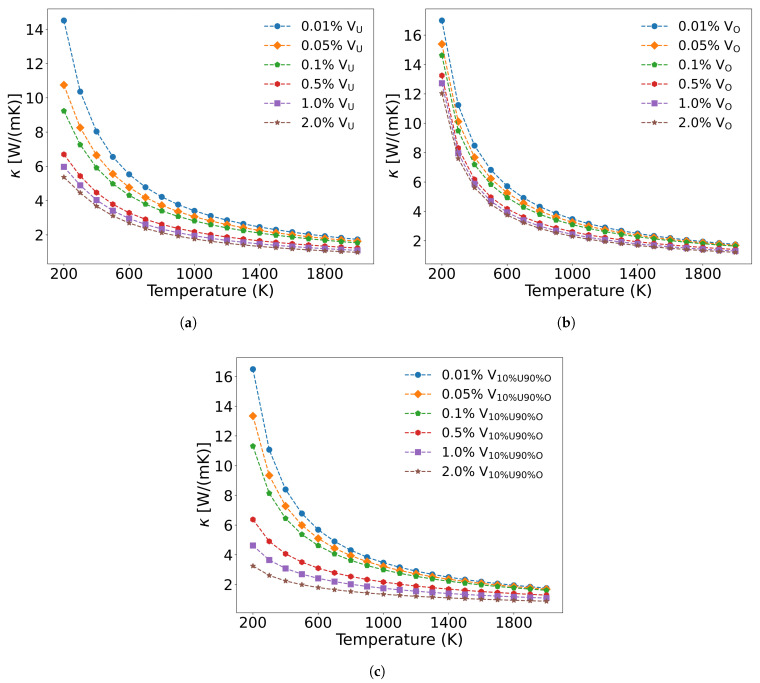
Thermal conductivity decrease in UO_2_ with different vacancy concentrations: (**a**) only uranium vacancies, (**b**) only oxygen vacancies, (**c**) Simultaneously containing uranium, and oxygen vacancies.

Unlike fission products, the concentration and distribution of irradiation-induced defects are strongly influenced by neutron energy spectra and irradiation temperature. The calculated formation energies of point defects in UO_2_ [59,60], revealing that the formation energy of oxygen interstitials is negative, and all other types of point defects exhibit positive and relatively high formation energies. According to Equation (9), since uranium interstitials and oxygen interstitials induce identical mass and radius differences (with the same proportionality constants being used), their scattering strength is equivalent to those of vacancies. To analyze the effect of irradiation defects on UO_2_ thermal conductivity, Figure 13a and b present the temperature-dependent thermal conductivity changes caused by irradiation-generated U and O vacancies. At equal concentrations, U vacancies demonstrate a greater impact on thermal conductivity reduction compared to O vacancies. However, in actual irradiated UO_2_, defects simultaneously contain both U and O vacancies. In this case, as shown in Figure 13c, both acoustic and optical phonon branches are scattered by vacancies, leading to more significant thermal conductivity degradation than when either U or O vacancies exist alone.

**Figure 14:**
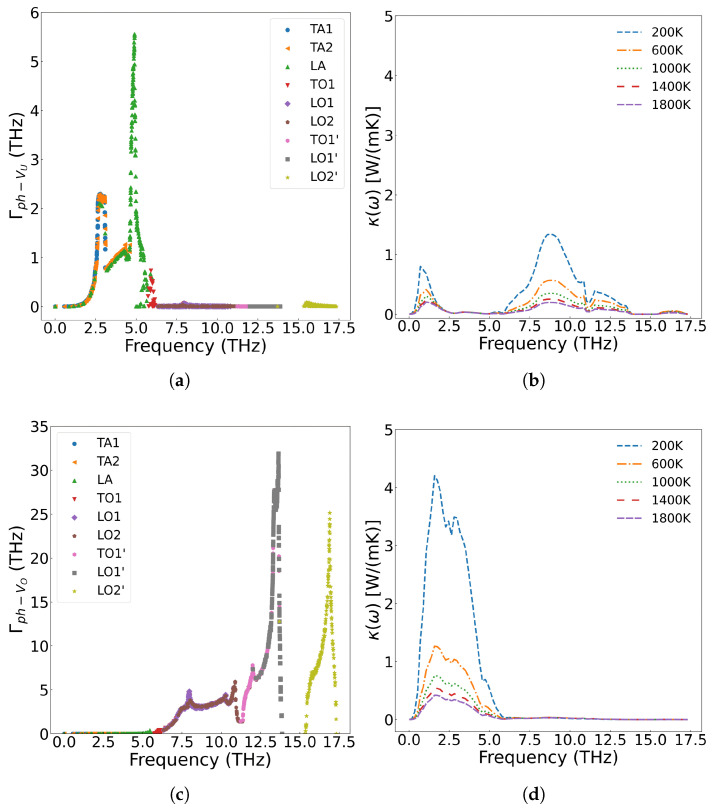

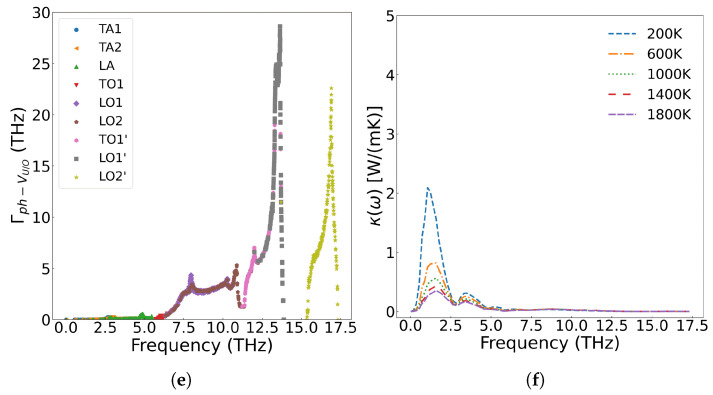
Frequency-dependent phonon scattering strength of 2.0 at% vacancies in UO_2_: (**a**) U vacancies only, (**c**) O vacancies only, (**e**) U and O vacancies; Effect on thermal conductivity from 2.0 at% vacancies in UO_2_: (**b**) U vacancies only, (**d**) O vacancies only, (**f**) U and O vacancies.

Kaloni et al. [61] employed the DFT+U method to investigate the electronic and phonon thermal conductivity of non-stoichiometric UO_x_ (x = 1.75–2.0, corresponding to oxygen vacancy concentrations of 0–12.5 at%). For x < 1.87 (oxygen vacancy concentration <6.5 at%), the phonon thermal conductivity decreases with increasing oxygen vacancy concentration, consistent with our computational results. While the electronic thermal conductivity in this regime exceeds that of stoichiometric UO_2_, the increase remains marginal. However, when the oxygen vacancy concentration exceeds 6.5 at%, the thermal conductivity of UO_x_ unexpectedly increases. This phenomenon arises from the insulator-to-metal transition in highly oxygen-deficient UO_x_, where enhanced electronic thermal conductivity dominates the overall heat transport. Figure 14 presents the scattering strength of 2.0 at% U and O vacancies on phonons and their temperature-dependent effects on thermal conductivity across different phonon frequencies. The results demonstrate that both U and O vacancies reduce thermal conductivity of UO_2_ but through distinct mechanisms: U vacancies primarily scatter low-frequency acoustic phonons, while O vacancies preferentially scatter high-frequency (greater than 5.8 THz) optical phonons. Notably, the combined presence of both vacancy types leads to broadband phonon scattering across all frequencies, resulting in more significant thermal conductivity reduction than either defect alone.

## 4. Conclusions

This study comprehensively investigated the phonon thermal conductivity of UO_2_ with defects using first-principles DFT+U calculations combined with the Boltzmann Transport Equation. The research elucidated the electronic structure, phonon spectrum, and thermal transport properties of UO_2_, with a focus on the effects of fission products and irradiation-induced defects. The key findings are summarized as follows:

1. Electronic and Phonon Properties: DFT+U calculations (U = 3.6 eV) accurately reproduced the Mott insulating nature of UO_2_, with a bandgap of 1.990 eV, consistent with experimental values (~2.0 eV). The phonon spectrum and Gruneisen parameter (γ = 1.86) aligned well with inelastic neutron scattering data, though high-frequency optical modes exhibited slight deviations due to negligible anharmonic effects at 0 K;
2. Impact of Fission Products: Metallic fission products (e.g., Mo, Ru, Ba) preferentially scattered low-frequency (less than 5.8 THz) phonons, reducing thermal conductivity by over 50% at high concentrations (76 GWd/tU). The scattering strength was governed by mass contrast and ionic radius mismatch, with the latter playing a dominant role. Fission gas Xe exhibited dual scattering mechanisms: occupying U vacancies primarily affected low-frequency phonons, while simultaneous occupation of U and O vacancies scattered both low- and high-frequency phonons, leading to more pronounced thermal conductivity degradation.
3. Irradiation-Induced Defects: U vacancies strongly scattered low-frequency phonons, whereas O vacancies targeted high-frequency (greater than 5.8 THz) modes. The combined presence of both vacancy types caused broadband phonon scattering, further reducing thermal conductivity. Notably, defect clusters and extended defects (not explicitly modeled here) likely introduce additional scattering channels in real irradiated fuels.

Although our results validate and extend prior theoretical and experimental work on thermal conductivity of irradiated UO_2_, there still exist some questions regarding defect-phonon interactions and thermal conductivity degradation mechanisms in defected UO_2_. For example, our defect models assume isolated point defects in irradiated fuels, but complex defect clusters (e.g., Xe bubbles, metal precipitates) and extended defects (dislocations, grain boundaries) likely introduce additional phonon scattering mechanisms not taken into consideration here. Future work should integrate DFT+U calculations with multiscale modeling approaches (e.g., phase-field, kinetic Monte Carlo) to simulate defect evolution under irradiation and discuss its impact on thermal transport. This study provides a fundamental understanding for thermal transport of UO_2_, emphasizes the critical role of defect management in mitigating thermal conductivity degradation, and helps to establish the design principle for more efficient nuclear fuels’ development.

## Figures and Tables

**Table 1:** Content, oxidation states, and ionic radii of fission elements in SIMFUEL samples of 76 GWd/tU burnup [54].

Fission Element	Ba	Ce	Cs	La	Mo	Sr	Y	Zr	Rh	Pd	Ru	Nd
Content (at%)	0.26	0.61	0.35	0.20	0.61	0.13	0.06	0.60	0.03	0.42	0.64	0.91
Oxidation State	2+	4+	1+	3+	4+	2+	3+	4+	0	0	0	3+
Ionic Radius (Å)	1.42	0.97	1.69	1.16	0.65	1.26	1.02	0.84	1.42	1.39	1.46	1.12

**Table 2:** Experimentally different concentrations of doping fission product element in simulated burnup UO_2_ samples [57].

Element	Concentrations (at%)		
	Sample1	Sample2	Sample3
Sr	0.232	0.362	0.466
Y	0.118	0.186	0.242
Zr	0.873	1.536	2.150
La	0.193	0.367	0.536
Ce	0.631	1.151	1.656
Nd	0.630	1.270	1.903

## Data Availability

The original contributions presented in this study are included in the article. Further inquiries can be directed to the corresponding author.

## Appendix A. Relaxation-Time Approximation

The scattering term on the right side of Equation (1) is expressed in the form of Equation (A1):

(A1) $$-{\left.\frac{\partial n_{q\nu }}{\partial t}\right|}_{scatt}=\sum_{q\nu }\Omega_{q\nu ,q^{\prime }\nu^{\prime }}\delta n_{q\nu }$$

where $\Omega_{q\nu ,q^{\prime }\nu^{\prime }}$ is the scattering matrix, and $\delta n_{q\nu }=n_{q\nu }-{\bar{n}}_{q\nu }$ represents the deviation of the phonon population from its equilibrium value. Using perturbation theory to solve the system under a small temperature gradient ∇T, the perturbed phonon population $n_{q\nu }$ is replaced by ${\bar{n}}_{q\nu }$. A function $f_{q\nu }$ is introduced to quantify the deviation of the phonon population in mode qν from the equilibrium distribution. Expanding the phonon distribution under the temperature gradient around the equilibrium distribution and retaining only the linear term [22], as shown in Equation (A2).

(A2) $$\begin{array}{cc} n_{q\nu } & =\frac{1}{\exp\left(\hbar \omega_{q\nu }/k_{B}T-f_{q\nu }\right)-1}\\ \; & ≃{\bar{n}}_{q\nu }-f_{q\nu }\frac{\partial {\bar{n}}_{q\nu }}{\partial (\hbar \omega_{q\nu }/k_{B}T)}\\ \; & ={\bar{n}}_{q\nu }+f_{q\nu }{\bar{n}}_{q\nu }\left({\bar{n}}_{q\nu }+1\right) \end{array}$$

For computational convenience, let $A_{q\nu ,q^{\prime }\nu^{\prime }}=\Omega_{q\nu ,q^{\prime }\nu^{\prime }}{\bar{n}}_{q^{\prime }\nu^{\prime }}\left({\bar{n}}_{q^{\prime }\nu^{\prime }}+1\right)$. The scattering matrix *A* is expressed as:

(A3) $$A_{q\nu ,q^{\prime }\nu^{\prime }}=\sum_{q^{\prime }\nu^{\prime },q^{\prime \prime }\nu^{\prime \prime }}\left[P_{q\nu ,q^{\prime }\nu^{\prime }}^{q^{\prime \prime }\nu^{\prime \prime }}+\frac{1}{2}P_{q\nu }^{q^{\prime }\nu^{\prime },q^{\prime \prime }\nu^{\prime \prime }}\right]+\sum_{q^{\prime }\nu^{\prime }}P_{q\nu ,q^{\prime }\nu^{\prime }}^{impurity}+P_{q\nu }^{others}$$

Based on Equations (A1)–(A3), the Boltzmann transport equation (BTE) is derived as:

(A4) $$\begin{array}{cc} -v_{q\nu }\frac{\partial {\bar{n}}_{q\nu }}{\partial T}\nabla T & =\sum_{q^{\prime }\nu^{\prime },q^{\prime \prime }\nu^{\prime \prime }}[P_{q\nu ,q^{\prime }\nu^{\prime }}^{q^{\prime \prime }\nu^{\prime \prime }}\left(f_{q\nu }+f_{q^{\prime }\nu^{\prime }}-f_{q^{\prime \prime }\nu^{\prime \prime }}\right)\\ \; & \;+\frac{1}{2}P_{q\nu }^{q^{\prime }\nu^{\prime },q^{\prime \prime }\nu^{\prime \prime }}\left(f_{q\nu }-f_{q^{\prime }\nu^{\prime }}+f_{q^{\prime \prime }\nu^{\prime \prime }}\right)]\\ \; & \;+\sum_{q^{\prime }\nu^{\prime }}P_{q\nu ,q^{\prime }\nu^{\prime }}^{impurity}\left(f_{q\nu }-f_{q^{\prime }\nu^{\prime }}\right)\\ \; & \;+P_{q\nu }^{others}f_{q\nu } \end{array}$$

Taking the derivative of ${\bar{n}}_{q\nu }$ with respect to temperature *T*, we obtain:

(A5) $$\frac{\partial {\bar{n}}_{q\nu }}{\partial T}=\frac{\hbar \omega_{q\nu }}{k_{B}T^{2}}{\bar{n}}_{q\nu }\left({\bar{n}}_{q\nu }+1\right)$$

The BTE can be written in matrix form as:

(A6) X=Pf

In X, the $X_{q_{\nu }}=-\frac{\hbar \omega_{q\nu }}{k_{B}T^{2}}{\bar{n}}_{q\nu }\left({\bar{n}}_{q\nu }+1\right)v_{q\nu }\nabla T$ , according to the definition of thermal conductivity:

(A7) Q=−κ∇T,

where κ is the thermal conductivity, and the heat flux *Q* is given by:

(A8) $$\begin{array}{c} Q=\frac{1}{N_{0}V}\sum_{q\nu }\hbar \omega_{q\nu }\Delta n_{q\nu }v_{q\nu }=\frac{1}{N_{0}V}\sum_{q\nu }\hbar \omega_{q\nu }{\bar{n}}_{q\nu }\left({\bar{n}}_{q\nu }+1\right)v_{q\nu }f_{q\nu } \end{array}$$

Here, $\Delta n_{q\nu }=n_{q\nu }-{\bar{n}}_{q\nu }$ represents the deviation from the equilibrium concentration, expressing the thermal conductivity κ as:

(A9) $$\kappa =-\frac{Q}{\nabla T}=-\frac{k_{B}T^{2}}{N_{0}V{\left|\nabla T\right|}^{2}}\sum_{q\nu }X_{q\nu }f_{q\nu }=\frac{k_{B}T^{2}}{N_{0}V{\left|\nabla T\right|}^{2}}Xf$$

The phonon scattering matrix P is expressed as P=Γ+Λ, where Γ is the diagonal scattering matrix and Λ is the off-diagonal matrix. Under the single-mode relaxation time approximation (SMRTA), only the diagonal elements of the scattering matrix are considered, and the off-diagonal elements (i.e., interactions between scatterings) are neglected, yielding P=Γ.

(A10) $${\left(Pf\right)}_{q\nu }≃P_{q\nu }f_{q\nu }=\Gamma_{q\nu }f_{q\nu }=-{\left.\frac{\partial n_{q\nu }}{\partial t}\right|}_{scatt}=\frac{n_{q\nu }-{\bar{n}}_{q\nu }}{\tau_{q\nu }}=\frac{{\bar{n}}_{q\nu }\left({\bar{n}}_{q\nu }+1\right)f_{q\nu }}{\tau_{q\nu }}$$

From the above, we derive:

(A11) $$\Gamma_{q\nu }=\frac{{\bar{n}}_{q\nu }\left({\bar{n}}_{q\nu }+1\right)}{\tau_{q\nu }}$$

Using Equation (A9)–(A11), the expression for the thermal conductivity κ [22] is:

(A12) $$\begin{array}{cc} \kappa & =\frac{k_{B}T^{2}}{N_{0}V{\left|\nabla T\right|}^{2}}Xf=\frac{k_{B}T^{2}}{N_{0}V{\left|\nabla T\right|}^{2}}XP^{-1}X\\ \; & =\frac{k_{B}T^{2}}{N_{0}V{\left|\nabla T\right|}^{2}}X\Gamma^{-1}X\\ \; & =\frac{\hbar^{2}}{N_{0}Vk_{B}T^{2}}\sum_{q\nu }v_{q\nu }^{2}\omega_{q\nu }^{2}{\bar{n}}_{q\nu }\left({\bar{n}}_{q\nu }+1\right)\tau_{q\nu } \end{array}$$
